# Potential nonpharmacological interventions to prevent frailty among elderly in low- and middle-income countries: A protocol for systematic review

**DOI:** 10.1097/MD.0000000000028708

**Published:** 2022-01-28

**Authors:** Rifat Ara, Umme Kulsum Monisha, Tashnova Jahan Nova, Sreshtha Chowdhury, Mohammad Hayatun Nabi, Mohammad Delwer Hossain Hawlader

**Affiliations:** a Department of Public Health, North South University, Dhaka, Bangladesh; b Infectious Disease Division, icddr,b, Dhaka, Bangladesh.

**Keywords:** elderly, frailty, low- and middle-income countries, nonpharmacological Interventions, prevention

## Abstract

**Background:**

Frailty syndrome is a medical condition defined by a progressive loss of function that usually begins beyond 65 and necessitates assistance with daily activities. There are both pharmacological and nonpharmacological approaches to prevent frailty. The purpose of this systematic review is to investigate viable nonpharmacological therapies for reducing frailty among the elderly in low- and middle-income countries, to develop an appropriate guideline to determine the applicability of these nonpharmacological interventions in various feasible settings.

**Methods:**

Two independent researchers will explore 5 electronic databases for relevant and promising studies. The selected articles will be subjected to a full-text examination following the initial screening. Two independent authors will analyze the risk of bias using the Cochrane risk of bias assessment tool. The review findings on various nonpharmacological approaches to prevent frailty will be presented as a narrative synthesis. There will be a sensitivity analysis and an assessment of study heterogeneity if possible.

**Results and conclusion:**

The systematic review protocol has been evaluated and approved by the institutional review board of North South University. The preferred reporting items for systematic review and meta-analysis protocol recommendations for precisely reporting health care interventions and the Cochrane group standards will be strictly followed in this systematic review protocol.

**PROSPERO Registration number:**

CRD42021290417

## 1 Introduction

During one's life, various biological deficiencies develop, which cause the body's homeostatic equilibrium to be disrupted.^[1–3]^ This process begins during a person's first year in the womb. It progresses at a varied rate and on various pathways depending on the individual, resulting in varying degrees of reduced resistance to internal and external stresses. The term “frailty” describes the predisposition of biologically aged adults to have unfavorable outcomes and undergo fast changes in health status. The development of chronic illnesses has been identified as a factor in the development of frailty.^[2]^ Frailty syndrome is a medical illness characterized by a progressive loss of function, which generally occurs beyond the age of 65 and results in help in carrying out daily living tasks. In addition, as the population becomes older, the need for specialized geriatric care will skyrocket, resulting in a significant rise in the demand for specialized services for the maintenance of elderly individuals.^[4]^

The development of illnesses may be thought of as the accumulation of distinct biological impairments that manifest as specific nosological phenomena when they reach a certain threshold.^[5,6]^ A condition known as “multimorbidity” (having 2 or more illnesses) develops as we get older. It is less prevalent in those younger than 65 years old and varies between 55% and 98% in people older than 65 years old; this condition is referred to as “aging-related multimorbidity”.^[7,8]^ Multimorbidity is a common and debilitating condition that affects the quality of life (QoL), raises medical costs, and prolongs the lives of those who suffer from the state. As chronic illnesses interact with one other, their adverse effects are magnified, and new clinical phenotypes are formed, which we would not predict from the sum of their impacts. Several factors contribute to multimorbidity, including an increase in global vulnerability and a decrease in resistance.^[5]^

According to a previous study, frail older persons are more at venture for unfavorable health consequences such as falls, mobility impairment, hospitalization, institutionalization, and an increased risk of mortality.^[9]^ According to recent findings, frailty may be reversible in some instances.^[10]^ Identifying frailty is thus critical in order to prevent, mitigate, and forestall deleterious health outcomes in older persons and their caregivers.

Frailty is also a significant concern in low- and middle-income countries (LMICs). In these areas where health resources are limited, a lack of equipment, qualified personnel, or adequate, validated evaluation techniques may impede examinations and treatments. As the world's populations age, the number of frail individuals grows, with LMICs bearing a disproportionate share of the burden.^[11]^ Nonpharmacological interventions (eg, multidisciplinary interventions, lifestyle modification, educational interventions, and so on) are alternatives that may be employed in older persons. Short physical performance battery, ADL (activities of daily life), nutritional markers, functional capacity, and Mini Nutritional Assessment are some of the most widely employed nonpharmacological approaches.

There are several studies of nonpharmacological therapies for the prevention and treatment of frailty in older patients. However, geriatric specialists in LMICs require a synthesized, methodologically sound document to guide their decision-making.^[12,13]^ As a result, this study aims to search the viable nonpharmacological therapies for minimizing frailty among the elderly in LMICs so that an appropriate guideline can be created to determine the applicability of these nonpharmacological interventions in different feasible settings. Approaches with the best outcome may have the potential for widespread application in geriatric healthcare services to improve the QoL of older communities in the low- and middle-income territories.

## 2 Methods and materials

### 2.1 Protocol

This systematic review protocol adheres to the standard criterion of systematic reviews of PRISMA-P (preferred reporting items for systematic review and meta-analysis protocol) guidelines for precisely reporting health care interventions and the Cochrane group standards.^[14,15]^ It is registered with the International prospective register of systematic reviews- PROSPERO, and the registration number is CRD42021290417. The PRISMA-P checklist for systematic review protocol has been attached as a supplementary file.

### 2.2 Eligibility criteria

The studies will be considered based on the criteria outlined below.

### 2.3 Participants

This review will consider the prefrail or frail older population aged 65 years or above from LMICs defined by the World Bank regardless of gender, region, or socio-economic status.^[16]^ However, the population residing outside LMICs will be factored out from this consideration.

### 2.4 Interventions

This review will include all interventional studies evaluating the effectiveness of nonpharmacological strategies to prevent frailty, such as lifestyle modification, nutritional advice, multi-component exercise, psychotherapy, health education, cognitive interventions, psychosocial interventions or psychotherapy, dietary supplementation, and any combination of the above interventions will be used as approaches.

### 2.5 Comparators

A comparison will be made between nonpharmacological interventions or no intervention control groups.

### 2.6 Outcomes

#### 2.6.1 Primary outcome

The primary outcome of this systematic review is the improvement or minimization of frailty ensuing the nonpharmacological interventions. We will look for a clear consensus since not enough information establishes the theory frailty. However, various metrics have been investigated and linked with improving frailty. Some of the most common are short physical performance battery, ADL, nutritional markers, functional capacity, and Mini Nutritional Assessment, which will be assessed as the initial outcome in this review.

#### 2.6.2 Secondary outcomes

The secondary outcomes will be evaluated if the included nonpharmacological approaches analyze some important scales that are often used to measure the improvement of frailty. Such measurements are often done by Katz 6 score, instrumental activities of daily living, grip strength, timed up and go test, gait speed, Geriatric Depression Scale, dietary variety score, and QoL.

### 2.7 Study design

This systematic review will include randomized controlled trials and quasi-experimental studies to illustrate the efficiency of available interventions for minimizing frailty. Other types of studies such as descriptive and analytical observational studies, pretest–posttest controlled studies, prospective cohort studies, case-control studies, and cross-sectional studies will be ruled out.

### 2.8 Setting

There will be no limitations in terms of study settings. Studies, which have been taken place in hospitals, geriatric healthcare service centers, or community settings will be within the scope of this review.

### 2.9 Exclusion criteria

Studies from developed, high-income countries or other than the list of LMICs by the World Bank will be excluded. However, if any article comprises data from both the high income countries and the LMICs, only the LMICs will be included and analyzed. Researches conducted on people under 65 years will be disqualified. This review will also eliminate the studies on pharmacological or surgical intervention or combining these approaches with nonpharmacological interventions. Moreover, researches published in any language other than English will be ruled out. In addition, this review will reject any other observational studies, other systematic reviews, ongoing trials, trial protocols, ongoing reviews, research letters or correspondences, editorials, conference papers, brief reports, periodicals, books, and book chapters.

### 2.10 Information sources

The following electronic bibliographic databases will be searched systematically for relevant articles using a detailed search strategy. These are MEDLINE, Web of Science, The Cochrane Library (Cochrane Central Register of Controlled Trials), Scopus, and Clinical Trials.gov.

### 2.11 Search strategy

A comprehensive and systematic search strategy for the MEDLINE or PubMed database will be designed using the keywords and “MeSH” terms according to population, intervention, comparison, and outcome. Later, this will be customized for additional bibliographical databases following the database-specific filters for clinical trials. Wild cards and truncations will be applied in search words as needed. The terminologies will be integrated using the Cochrane MEDLINE filter for controlled trials of interventions. The keywords of the search strings will be correlated to the population, interventions, and desired outcomes. According to population, intervention, comparison, and outcome, the essential terms implemented in this review are listed in Table 1.

**Table 1:** Key terms used for developing a comprehensive search strategy.

Population (P)	Intervention (I)	Comparison (C)	Outcome (O)
“Elderly”	“Acupressure”	“Effectiveness”	“Frailty”
“Older adults”	“Exercise”	“Usefulness”	“Frailties”
“65+ yr”	“Resistance exercise”	“Efficiency”	“Frailness”
“65 yr or above”	“Home-based exercise”	“Potency”	“Frailty syndrome”
“Over 65 yr”	“Multi-component exercise”	“Efficacy”	“Debility”
“Frail”	“Physical activity”		“Debilities”
“Prefrail”	“Physical interventions”		
“Frail older”	“Lifestyle advice”		
“Frail elderly”	“Nutritional advice”		
“Elderly frail”	“Nutritional interventions”		
	“Mediterranean diet”		
	“Memory training”		
	“Cognitive interventions”		
	“Psychosocial interventions”		
	“Psychotherapy”		
	“Aromatherapy”		
	“Health education”		

The search period will be between January 2000 and the date of the searches. Before the final analysis, a final search will be conducted to retrieve further potential studies for inclusion. Table 2 illustrates the comprehensive search strategy using the keywords and “MeSH” terms for MEDLINE.

**Table 2:** Comprehensive search strategy.

Search strategy: PubMed format	
#1	“aged”[MeSH Terms] or “aged”[All Fields] or “elderly”[All Fields] or “elderlies”[All Fields] or “elderly s”[All Fields] or “elderlys”[All Fields] or (“older”[All Fields] and “adults”[All Fields]) or “older adults”[All Fields] or (“65”[All Fields] and “years”[All Fields]) or (“above”[All Fields] and “65”[All Fields] or “years”[All Fields]) or (“Over”[All Fields] and “65”[All Fields] or “years”[All Fields]) or “frail”[All Fields] or “frails”[All Fields] or “Prefrail”[All Fields] or “frail older”[All Fields] or (“frail elderly”[MeSH Terms] or (“frail”[All Fields] and “elderly”[All Fields]) or “frail elderly”[All Fields]) or (“elderly”[All Fields] and “frail”[All Fields]) or “elderly frail”[All Fields]
#2	“acupressure”[MeSH Terms] or “acupressure”[All Fields] or “exercise”[MeSH Terms] or “exercise”[All Fields] or “exercises”[All Fields] or “exercise therapy”[MeSH Terms] or “exercise therapy”[All Fields] or “resistance exercise”[All Fields] or “Home-based exercise”[All Fields] or “Multi-component exercise”[All Fields] or (“physical”[All Fields] and “activity”[All Fields]) or “physical activity”[All Fields] or “physical intervention”[All Fields] or “physical interventions”[All Fields] or “life style”[MeSH Terms] or “lifestyle advice”[All Fields] or “lifestyle advices”[All Fields] or “nutritional advice”[All Fields] or “nutritional advices”[All Fields] or (“diet therapy”[MeSH Terms] or (“diet”[All Fields] and “therapy”[All Fields]) or “diet therapy”[All Fields] or (“nutritional”[All Fields] and “interventions”[All Fields]) or “nutritional interventions”[All Fields]) or (“diet, mediterranean”[MeSH Terms] or “mediterranean diet”[All Fields]) or (“memory”[All Fields] and “training”[All Fields]) or “memory training”[All Fields] or ((“cognitive”[All Fields] or “cognitives”[All Fields]) and (“interventions”[All Fields] or “interventive”[All Fields] or “methods”[MeSH Terms] or “methods”[All Fields] or “intervention”[All Fields] or “interventional”[All Fields])) or (“psychosocial intervention”[MeSH Terms] or (“psychosocial”[All Fields] and “intervention”[All Fields]) or “psychosocial intervention”[All Fields] or “psychosocial interventions”[All Fields]) or (“psychotherapy”[MeSH Terms] or “psychotherapy”[All Fields] or “psychotherapies”[All Fields] or “psychotherapy s”[All Fields]) or (“aromatherapy”[MeSH Terms] or “aromatherapy”[All Fields] or “aromatherapies”[All Fields]) or (“health education”[MeSH Terms] or (“health”[All Fields] and “education”[All Fields]) or “health education”[All Fields])
#3	“effect”[All Fields] or “effecting”[All Fields] or “effective”[All Fields] or “effectively”[All Fields] or “effectiveness”[All Fields] or “effectivenesses”[All Fields] or “effectives”[All Fields] or “effectivities”[All Fields] or “effectivity”[All Fields] or “effects”[All Fields] or “useful”[All Fields] or “usefulness”[All Fields] or “efficiences”[All Fields] or “efficiency”[MeSH Terms] or “efficiency”[All Fields] or “efficiencies”[All Fields] or “efficient”[All Fields] or “efficiently”[All Fields] or “efficients”[All Fields] or “potencies”[All Fields] or “potency”[All Fields] or “efficacies”[All Fields] or “efficacious”[All Fields] or “efficaciously”[All Fields] or “efficaciousness”[All Fields] or “efficacy”[All Fields]
#4	“frailty”[MeSH Terms] or “frailty”[All Fields] or “frailty/classification”[MeSH Terms] or “frailty/complications”[MeSH Terms] or “frailty/diet therapy”[MeSH Terms] or “frailty/epidemiology”[MeSH Terms] or “frailty/nursing”[MeSH Terms] or “frailty/prevention and control”[MeSH Terms] or “frailty/psychology”[MeSH Terms] or “frailty/radiotherapy”[MeSH Terms] or “frailty/rehabilitation”[MeSH Terms] or “frailty/therapy”[MeSH Terms] or “frailties”[All Fields] or “frailness”[All Fields] or (“frailty”[All Fields] and “syndrome” [All Fields]) or “frailty syndrome” [All Fields] or “debility”[All Fields] or “debilities” [All Fields]
#5	#1 and #2 and #3 and #4
	From 2000
	English language paper

### 2.12 Study records

#### 2.12.1 Data management

Articles retrieved from the literature searches in different databases will be organized by the reference management software Rayyan Qatar Computing Research Institute. All the search results will be compiled into a single library, where duplicate articles will be identified and excluded. The reviewers will check the remaining articles for screening.

#### 2.12.2 Selection process

Following the inclusion and exclusion criteria, 2 review authors will skim the title and abstract of the included articles independently to screen the potential studies. After title and abstract screening, a complete text evaluation of the presumed articles will be done. Afterward, the selected studies will be evaluated further for final inclusion. A third independent reviewer will settle any disagreement through discussion with the previous 2. Multiple publications and the reasons for excluding researches will be reported. The PRISMA flow diagram will demonstrate the process of study inclusion and exclusion.^[17]^

#### 2.12.3 Data extraction

Two reviewers will independently extract data using a standardized Microsoft Excel (The Microsoft Corporation) Sheet to record the following information: the study population, study settings, baseline characteristics of study participants, study methods, details of the interventions for frailty prevention, recruitment process, attrition rates, primary and secondary outcomes, periods of measurement, and information regarding the risk of bias (ROB). If a disagreement emerges between 2 reviewers, it will be settled through discussion while seeking the third reviewer's opinion if necessary.

#### 2.12.4 Risk of bias assessment

Two researchers will apply the Cochrane ROB assessment tool to evaluate the ROB independently of the included randomized controlled trials.^[18]^ This evaluation will be performed based on the following domains: randomization, allocation concealment, blinding at various stages, incomplete data, and reporting of any additional bias. According to the review authors’ assessments, the studies will be categorized as low ROB, high ROB, or uncertain. Thorough discussions will resolve any discord between the reviewers. However, insights from a third researcher will be pursued if necessary.

#### 2.12.5 Strategy for data synthesis

We will assess the current interventions, map them out, and summarize the studies’ baseline features following the full-text screening of the studies. A narrative synthesis will assist in comparing the resemblances and differences of the studies, emphasizing the population, interventions, and outcomes. Data will be pooled if heterogeneity measured by I^2^ value is found less than 50%. Standardized mean differences will be used to combine improvements in the frailty index scores following the intervention. Risk ratios will be used to compare proportions such as the prevalence of frailty between the intervention and control groups. A random-effects model will be utilized for data synthesis to account for any heterogeneity. If possible, a sensitivity analysis will be performed to acquire an in-depth view of the study findings. Subgroup analysis for variables such as ethnicity, sex, and presence of comorbidities will be performed if appropriate data are available and incorporated in meta-regression models to assess the effect of participant's demographic aspects on the potency of the interventions. We will also look for publication bias by developing a funnel plot using the Review Manager software (Cochrane).

## 3 Discussion

In the last 2 decades, global life expectancy has risen from 66.8 to 73.3 years, with healthy life expectancy rising from 58.3 to 63.7 years. Surprisingly, LMICs are making the most progress, owing to prompt lowering in child mortality and communicable diseases.^[19]^ By 2050, the proportion of the population aged 65 and above (15.9%) will be more than twice that of children under the age of 5 (7.1%) and will also overtake that of young adults aged between 15 and 24 (13.7%).^[20]^ Such demographic transition to the aging population might invite health challenges such as multimorbidity, and complex care needs to defend the form of frailty. The deterioration of physical and functional capacities will also create service accessibility barriers, demanding more attention in delivering health care services to the frail elderly.

In LMICs, The World Health Organization study on global aging and adult health indicated variable rates of self-reported unfilled health care requirements among those aged 65 and older.^[21]^ In this setting, prevention of frailty is critical in preparing healthcare systems to respond to people's desires to maintain autonomy as they age. Some earlier reviews have evaluated frailty in developing countries, but this is the first to look exclusively at nonpharmacologic interventions for frailty prevention in LMICs. Moreover, this systematic review is strongly aligned with the sustainable development goals, as through prevention and treatment, sustainable development goal 3.4 aspires to reduce premature mortality from noncommunicable diseases (NCDs) by one-third and increase mental health and well-being.^[22]^

Out-of-pocket expenditures for NCDs are rapidly increasing in LMICs. Identifying effective nonpharmacological therapies will significantly reduce the incidence and progression of frailty. Consequently, the burden of NCDs and their impact on the economy will be reduced in LMICs. Therefore, the systematic review will look at all of the nonpharmacological interventions available and feasible in the settings of LMICs with the aim to recommend the effective ones that have a significant impact on preventing frailty. We believe this review will also help to discuss future practice and research implications. Governments, healthcare providers, and funding organizations may be able to use the accumulated empirical data of accessible LMIC-based nonpharmacological therapies to help them decide how to devote future donations to the most beneficial strategy in similar circumstances. In addition, responsible authorities could choose cost-effective nonpharmacological interventions over the current pharmacological one, based on the effectiveness.

The review's merits include a clearly stated aim, a systematic and transparent process. The screening and extraction will be carried out by 2 researchers using pretested and standardized extraction forms. In addition, each study's quality will be assessed and shown in tabulation form. To our knowledge, the protocol's primary limitation is that it only evaluates publications written in English. If there is any research published in Chinese, Latin, or other languages, we may miss it. Despite a predefined systematic approach, the study will include assessments made by review authors, leading to bias.

## Acknowledgments

The authors would like to acknowledge the technical and training contribution of the Public Health Professional Development Society and the Governments of Bangladesh, Canada, Sweden, and the UK for providing unrestricted support to icddr,b research efforts.

## Author contributions

**Conceptualization:** Rifat Ara, Umme Kulsum Monisha, Tashnova Jahan Nova, Sreshtha Chowdhury, Mohammad Hayatun Nabi, Mohammad Delwer Hossain Hawlader.

**Data curation:** Rifat Ara, Umme Kulsum Monisha, Tashnova Jahan Nova, Sreshtha Chowdhury.

**Formal analysis:** Rifat Ara, Umme Kulsum Monisha, Tashnova Jahan Nova, Sreshtha Chowdhury, Mohammad Delwer Hossain Hawlader.

**Funding acquisition:** Mohammad Delwer Hossain Hawlader.

**Investigation:** Mohammad Hayatun Nabi, Mohammad Delwer Hossain Hawlader.

**Methodology:** Rifat Ara, Umme Kulsum Monisha, Tashnova Jahan Nova, Sreshtha Chowdhury, Mohammad Hayatun Nabi, Mohammad Delwer Hossain Hawlader.

**Project administration:** Mohammad Hayatun Nabi, Mohammad Delwer Hossain Hawlader.

**Resources:** Mohammad Hayatun Nabi, Mohammad Delwer Hossain Hawlader.

**Software:** Rifat Ara, Umme Kulsum Monisha, Tashnova Jahan Nova, Sreshtha Chowdhury.

**Supervision:** Rifat Ara, Mohammad Hayatun Nabi, Mohammad Delwer Hossain Hawlader.

**Validation:** Rifat Ara, Umme Kulsum Monisha, Tashnova Jahan Nova, Sreshtha Chowdhury, Mohammad Hayatun Nabi, Mohammad Delwer Hossain Hawlader.

**Visualization:** Mohammad Hayatun Nabi, Mohammad Delwer Hossain Hawlader.

**Writing – original draft:** Rifat Ara, Umme Kulsum Monisha, Tashnova Jahan Nova, Sreshtha Chowdhury.

**Writing – review & editing:** Rifat Ara, Mohammad Hayatun Nabi, Mohammad Delwer Hossain Hawlader.

## References

[1] Rockwood K Mitnitski A. Frailty in relation to the accumulation of deficits. J Gerontol Ser A Biol Sci Med Sci 2007;62:722–7. 17634318 10.1093/gerona/62.7.722

[2] Clegg A Young J Iliffe S Rikkert MO Rockwood K. Frailty in elderly people. Lancet 2013;381:752–62. 23395245 10.1016/S0140-6736(12)62167-9 PMC4098658

[3] Rodriguez-Mañas L Fried LP. Frailty in the clinical scenario. Lancet 2015;385:e7–9. 25468154 10.1016/S0140-6736(14)61595-6

[4] Morante JJH Martínez CG Morillas-Ruiz JM. Dietary factors associated with frailty in old adults: a review of nutritional interventions to prevent frailty development. Nutr 2019;11:102. 10.3390/nu11010102 PMC6356476 30621313

[5] Vetrano DL Calderón-Larrañaga A Marengoni A. An international perspective on chronic multimorbidity: approaching the elephant in the room. J Gerontol Ser A Biol Sci Med Sci 2018;73:1350–6. 28957993 10.1093/gerona/glx178 PMC6132114

[6] Fabbri E An Y Zoli M. Aging and the burden of multimorbidity: associations with inflammatory and anabolic hormonal biomarkers. J Gerontol Ser A Biol Sci Med Sci 2015;70:63–70. 25104822 10.1093/gerona/glu127 PMC4296167

[7] Calderón-Larrañaga A Vetrano DL Onder G. Assessing and measuring chronic multimorbidity in the older population: a proposal for its operationalization. J Gerontol Ser A Biol Sci Med Sci 2017;72:1417–23. 28003375 10.1093/gerona/glw233 PMC5861938

[8] Marengoni A Angleman S Melis R. Aging with multimorbidity: a systematic review of the literature. Ageing Res Rev 2011;10:430–9. 21402176 10.1016/j.arr.2011.03.003

[9] Fried LP Tangen CM Walston J. Frailty in older adults: evidence for a phenotype. J Gerontol Ser A Biol Sci Med Sci 2001;56:146–57. 10.1093/gerona/56.3.m146 11253156

[10] Ng TP Feng L Nyunt MSZ. Nutritional, physical, cognitive, and combination interventions and frailty reversal among older adults: a randomized controlled trial. Am J Med 2015;128:1225.e1–36.e1. 26159634 10.1016/j.amjmed.2015.06.017

[11] Gray WK Richardson J McGuire J. Frailty screening in low- and middle-income countries: a systematic review. J Am Geriatr Soc 2016;64:806–23. 27100577 10.1111/jgs.14069

[12] Tan PJ Khoo EM Chinna K Hill KD Poi PJH Tan MP. An individually-tailored multifactorial intervention program for older fallers in a middle-income developing country: Malaysian Falls Assessment and Intervention Trial (MyFAIT). BMC Geriatr 2014;14:78. 24951180 10.1186/1471-2318-14-78 PMC4080753

[13] Marcucci M Damanti S Germini F. Interventions to prevent, delay or reverse frailty in older people: a journey towards clinical guidelines. BMC Med 2019;17:193. 31660959 10.1186/s12916-019-1434-2 PMC6819620

[14] Shamseer L Moher D Clarke M. Preferred reporting items for systematic review and meta-analysis protocols (PRISMA-P) 2015: elaboration and explanation. BMJ 2015. 349. 10.1136/bmj.g7647 25555855

[15] Higgins JPT Thomas J Chandler J. Cochrane Handbook for Systematic Reviews of Interventions. 2nd ed. Chichester, UK: John Wiley & Sons; 2019.

[16] World Bank Group. World Bank Country and Lending Groups. Published 2020. Available at: https://datahelpdesk.worldbank.org/knowledgebase/articles/906519-world-bank-country-and-lending-groups. Accessed December 13, 2021.

[17] Moher D Liberati A Tetzlaff J. Preferred reporting items for systematic reviews and meta-analyses: the PRISMA statement. PLoS Med 2009;6:e1000097. 19621072 10.1371/journal.pmed.1000097 PMC2707599

[18] Cochrane Risk of Bias (ROB) Assessment Tool. Available at: https://methods.cochrane.org/bias/resources/rob-2-revised-cochrane-risk-bias-tool-randomized-trials. Accessed December 13, 2021.

[19] World Health Statistics 2021. Published 2021. Available at: https://apps.who.int/iris/bitstream/handle/10665/342703/9789240027053-eng.pdf. 18. Accessed December 13, 2021.

[20] World Population Prospects 2019: Highlights. Published 2019. Available at: https://www.un.org/development/desa/publications/world-population-prospects-2019-highlights.html. Accessed December 13, 2021.

[21] WHO Study on global AGEing and adult health (SAGE) [online database]. Published 2021. Available at: https://www.who.int/data/data-collection-tools/study-on-global-ageing-and-adult-health. Accessed December 13, 2021.

[22] United Nations. Sustainable Development Goals. Published 2021. Available at: https://sdgs.un.org/goals/goal3. Accessed December 13, 2021.

